# Influence of Low- to Medium-Kaolinite Clay on the Durability of Limestone Calcined Clay Cement (LC3) Concrete

**DOI:** 10.3390/ma16010374

**Published:** 2022-12-30

**Authors:** Kiran Ram, Matea Flegar, Marijana Serdar, Karen Scrivener

**Affiliations:** 1 Department of Materials, Faculty of Civil Engineering, University of Zagreb, 10000 Zagreb, Croatia; 2 Laboratory of Construction Materials, École Polytechnique Fédérale de Lausanne, 1015 Lausanne, Switzerland

**Keywords:** kaolinite calcined clay, chloride penetration, electrical resistivity, chloride binding, capillary pore volume, critical pore entry diameter

## Abstract

The kaolinite content is principally responsible for the durability performance of Limestone Calcined Clay Cement (LC3), which calls into question its global applicability. The clay supply has a significant impact on the LC3 system’s reduced carbon footprint advantage. The influence of kaolinite concentration from two separate clays (collected in East South-East Europe) on the durability performance of concrete was investigated in this study. The low-kaolinitic clay had 18% kaolinite, while the medium-kaolinitic clay contained around 41% kaolinite. The compressive strength, chloride intrusion, electrical conductivity, surface resistivity, and sorptivity index were measured on concrete after 28 days. Furthermore, the pore structure development of these mixtures was investigated in relation to the kaolinite content of the mixtures. The reactivity test was performed on clays to measure their reactivity levels within the cementitious system. The results show that kaolinite content has a moderate effect on compressive strength, but it has a considerable effect on other durability indices. When compared to the Portland cement mixture, the chloride migration and diffusion coefficients were reduced by 50% and 36%, respectively, in the combination with a medium kaolinite content (more than 40%). The low-kaolinitic clay, on the other hand, achieved 60% of the chloride penetration resistance of the medium-kaolinitic clay. Furthermore, low-kaolinitic clay has been demonstrated to be suitable for low-carbon concrete in moderate exposure conditions.

## 1. Introduction

The global climate has changed dramatically in recent decades. Since the pre-industrial era, global warming has led to a temperature increase of 1.1 °C [1]. It is widely known that industrial carbon emissions contribute significantly to global warming. Similar to other industries, the cement industry has implemented a variety of strategies to reduce its carbon footprint. Over the past several decades, low-carbon cements have received the most attention. The majority of low-carbon cements employ supplementary cementitious materials (SCMs), thereby reducing the clinker factor. The concrete industry aims to achieve a clinker factor of 0.52 by 2050, down from the current value of 0.62 [2]. Typically, fly ash, blast furnace slag, and natural pozzolana are employed in the production of low-carbon cements [3,4,5,6]. However, most of these materials are byproducts from industries which are also going through green transition. Fly ash, for example, is a byproduct from coal-fired power plants that are expected to close in the coming years [7]. If adequate replacement for the most common SCMs is not found, the transition to carbon neutrality will be delayed. Due to their widespread availability, kaolinitic clays become an important source of SCMs in this scenario [8]. Particularly, Limestone Calcined Clay Cement (LC3) has proven its capability to be a good replacement as a binder in concrete [5,6,7,8]. The LC3 system has better early-age strength, chloride ingress resistance, and a carbon footprint that is 40 percent lower than Portland cement [9,10,11,12,13,14,15,16,17,18,19].

In general, thermal activation of kaolinitic clays leads to the formation of metakaolin, which gives the clays their reactivity. In the LC3 system, metakaolin reacts with portlandite, water, and sulfate to form C-A-S-H, ettringite, and AFm phases [14,15]. Other clay minerals, such as illite, also contribute to pozzolanic reactivity; however, various researchers have reported that the benefits are negligible compared to kaolinite [16]. Therefore, the quantity of kaolinite within the clay is the determining factor for properties of LC3. Avet et al. reported that the amounts of reacted metakaolin in the LC3 system were quite similar for calcined clays with more than 50% kaolinite content and concluded that optimum kaolinite content is about 60 percent [17]. 

Clays with the recommended kaolinite cannot be found everywhere. Flegar et al. found that most of the clays collected in Croatia had no more than 20% kaolinite [20]. Few studies have been conducted on the use of low-grade clay in concrete, and it has been reported that low-grade clays could be used to replace cement [21,22,23,24]. In the long run, the phase assemblage of the LC3 system was found to be quite similar regardless of the different kaolinite content, while the kinetics of the evolution of hydration products were distinct [25]. Therefore, the clays with lower kaolinite content could also be a viable solution to make LC3 system.

The primary focus of this study was to analyze the performance of concrete with two different clays collected from East South-East Europe: one with a moderate amount of kaolinite and the other with a very low kaolin content. LC3 systems were formulated using these two clays to evaluate their durability in terms of electrical resistivity, chloride penetration, and water absorption. The aim of the research was to compare their durability and identify critical properties which are the most impacted by the kaolinite content. In addition, the effect of kaolinite content on the binding of chlorides and pore structure was systematically evaluated. 

## 2. Materials and Methods

### 2.1. Materials

**Figure 1:**
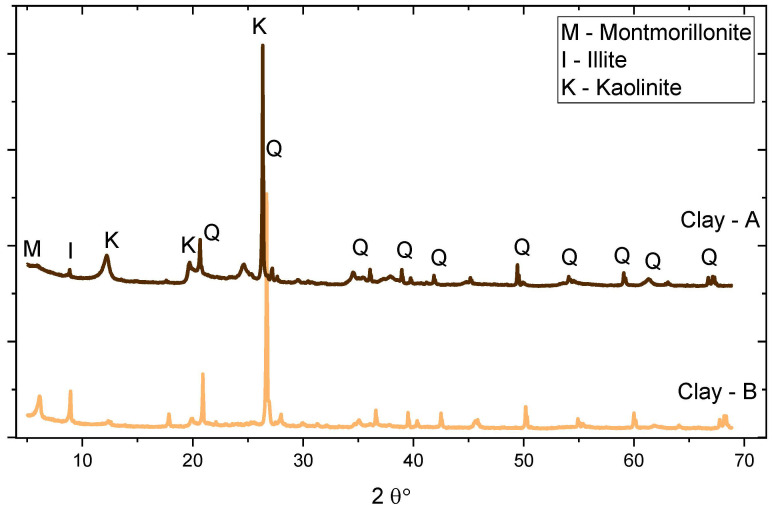
XRD pattern of raw clays.

**Figure 2:**
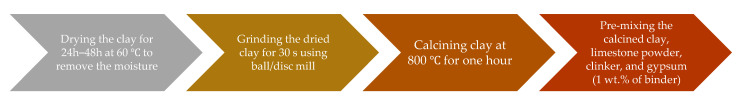
Steps involved in the preparation of LC3 in the lab for this study.

**Figure 3:**
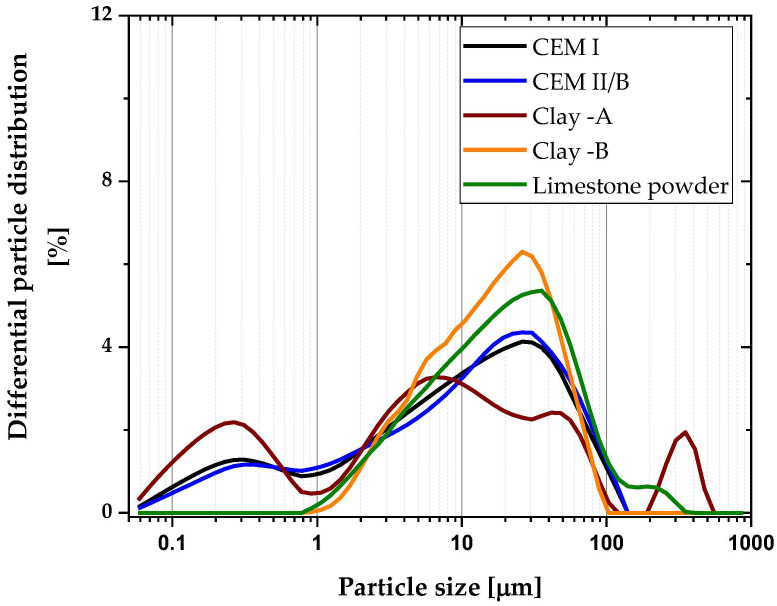
Particle size distribution of all materials used in this study.

Portland cement of type CEM I 42.5R (as per EN 196-1) was used in all mixtures as a primary binder. Two different clays were used, labeled A and B. The kaolinite of raw clays was determined using thermogravimetric (TG) analysis, and the mass loss between 400 °C and 600 °C was attributed to the dihydroxylation of kaolinite [16]. Limestone used in the study was obtained as waste powder from limestone quarry in Zvečaj. In addition, CEM II/B-S (blended cement with blast furnace slag) was utilized to make a mix for comparing the performance of limestone-calcined clay mixes to a classical blended cement available at the market. As shown in Table 1, the raw materials’ chemical oxide compositions were measured using X-ray fluorescence. In addition to oxides, the mineralogical composition of clinker and two clays was evaluated using X-ray diffraction technique (Table 2) and the XRD pattern illustrated in Figure 1. Following the whole process illustrated in Figure 2, clays were made as a binder. Figure 3 depicts the results of laser diffraction tests on the particle size distributions of all materials.

Three aggregate classes were utilized, including two coarse aggregate classes (16/8 and 8/4) and one fine aggregate class (0/4). Table 3 contains the physical properties of each aggregate fraction. In this study, the calcined clay to limestone powder ratio was taken at 2:1, according to several previous studies [8]. The mix proportions adopted in this study are listed in Table 4. LC3-A and LC3-B are the concrete mixtures using calcined clay A and B, respectively. Limestone calcined clay mixtures were prepared with CEM I as the primary binder.

In addition, a commercial superplasticizer (SP) with a solids content of 35% was used to ensure sufficient workability. Similarly, 1% gypsum was added to each mixture (in LC3 systems) of blended cement to prevent false settings produced by the system’s under-sulphation [26]. All mixes were made and verified for fresh properties according to EN 12350 specifications. Specimens were demolded twenty-four hours after casting and placed in the humidity chamber (relative humidity maintained at greater than 95% and temperature maintained at 20 °C) until the day of testing.

### 2.2. Test Methods

**Figure 4:**
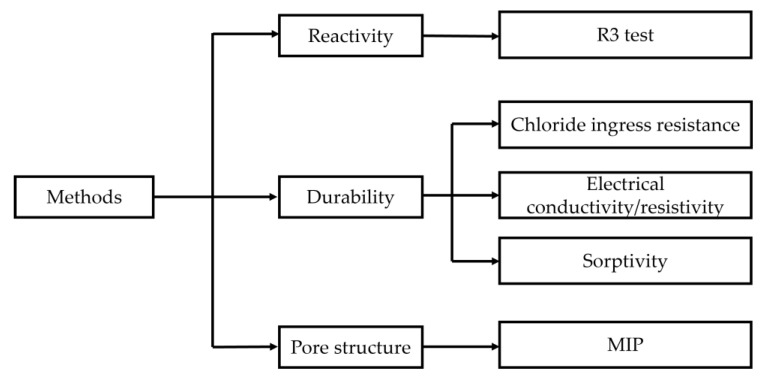
Layout of the experimental work.

The entire experimental program was divided into three categories: reactivity, durability properties, and pore structure development. The layout of the experimental plan is depicted in Figure 4. All the techniques are explained in the following section.

#### 2.2.1. Reactivity Test

A reactivity test was conducted using TAM Air isothermal calorimeter with 8 channels (TA Instruments, New Castle, DE, USA) as per ASTM C1897-20 [27] to determine the reactivity levels of clays with different kaolinite content. This test would give the pozzolanic reactivity of clays to isolate the clinker effect. Paste samples were prepared, which contained the clay (i.e., SCM), sulfate, and alkali, and placed into an isothermal calorimeter at a temperature of 40 °C for seven days. During these seven days, the total heat generated was monitored, indicating the reactivity of clay. The bound water content of these two clays were also measured as an indicator of reactivity. 

#### 2.2.2. Concrete Preparation and Compressive Strength

Clinker, calcined clay, limestone, and gypsum were manually mixed to obtain a homogenous mixture prior to concrete mixing. The highest capacity of the pan mixer used to mix the concrete was 70 L. The order of mixing was similar for each batch including the control mixture. The initial mixing of the dry components for two minutes was followed by the addition of 90% of the water and further mixing for two minutes. In the final phase, the superplasticizer was mixed with the remaining water for a further three minutes. In total, the mixing time met the requirement mentioned by the superplasticizer manufacturer to achieve the maximum dispersion of admixture. After mixing, the slump, wet density, temperature, and air content were measured immediately. Three concrete specimens of 15 cm × 15 cm × 15 cm were prepared to measure the compressive strength after 7 and 28 days of curing, and the test parameters and procedures followed the EN 12390-3 standard. 

#### 2.2.3. Chloride Ingress Resistance

Cylindrical specimens of 20 cm height and 10 cm diameter were prepared to test the chloride penetration resistance of the mixtures. After 28 days of curing, the cylindrical specimens were split diametrically into three slices with a thickness of 5 cm. Chloride penetration resistance was measured after 28 days of curing using Rapid Chloride Permeability Test—PROOVE´it (Germann Instruments, Copenhagen, Denmark), as per NT BUILD 492 [28]. Using this method, a non-steady-state migration coefficient (*D*_nssm_) is obtained, calculated by the Nernst-Plank equation expressed in Equation (1).

(1) $$D_{nssm}=\frac{RT}{zFE}.\frac{x_{d}-\alpha \surd x_{d}}{t}$$

where *x_d_* is the average measured depth of chloride penetration after the test duration, while other parameters were taken from the NT BUILD 492. 

Chloride diffusion coefficients of each were evaluated based on NT BUILD 443 [29], and the specimens were coated with epoxy resin on all sides except the bottom one for chloride exposure. After coating, the specimens were left another 24 h for the epoxy to fully dry and then fully immersed in 16.5% NaCl solution for 35 days. After 35 days, the profile grinding (depths were taken according to NT BUILD 443) was performed to obtain powder for chemical analysis. The total chloride content in the powder was determined using the potentiometric titration method described in standard EN 14629-2007 [30]. Thereafter, the effective chloride diffusion coefficients (*D*_e_)P were determined by fitting the total chloride contents in second Fick’s law according to NT BUILD 443,

(2) $$C\;\left(x,t\right)=C_{s}-\left(C_{s}-C_{i}\right).\;\mathrm{erf}\left(x|\sqrt{4D_{e}t}\right)$$

where *C*(*x*,*t*) is the measure of chloride content at depth (*x*) and exposure time (*t*). The values of apparent chloride diffusion coefficients could be extracted after fitting the measured chloride contents. The free chloride content of each depth was also determined as water-soluble chloride, according to ASTM C1218. After the determination of free chloride and total chloride content, the bound chloride was determined as a difference between the total and free chloride. 

#### 2.2.4. Electrical Conductivity/Surface Resistivity

The electrical conductivity of each mixture was determined after 28 days of curing according to ASTM C1760 [31]. The initial current values from the chloride migration test were used to calculate Equation (3).

(3) $$Conductivity,\;\sigma =1273.2\;.\frac{I_{i}}{V}.\frac{L}{D^{2}}$$

where current (*I*) is measured as the initial current by imposing voltage (*V*) to the specimen, and other parameters were taken from the standard.

The surface resistivity of each mixture was determined by Wenner’s four-probe resistivity meter on the sides of cylindrical specimens, which was used to determine the chloride transport coefficients [32]. The measurements were taken in saturated condition and at four different locations of each specimen. A total of 12 readings were taken, and the average was reported as surface resistivity.

#### 2.2.5. Sorptivity

Sorptivity, the rate of absorption in concrete, is commonly used to evaluate the resistance of concrete to moisture penetration via capillary absorption. This factor was identified as a major contributor to the initial penetration of chloride into the specimen [13], and this initial penetration has a significant impact on the subsequent movement of chloride and, consequently, the de-passivation of steel. In this study, the sorptivity of each sample was determined based on the South African Durability Index Manual [33]. After curing, the specimen was dried at 50 °C for seven days and then placed in a desiccator for four hours. After drying, specimens were placed on a tray which contained saturated Ca (OH)_2_ solution. The specimens were supported by wooden rollers, and the solution level was restricted to within 2 mm of the surface. The specimen’s weight was measured after 3, 5, 9, 12, 16, 20, and 25 min. After weighing the samples, they were conditioned for one day in a saturated calcium hydroxide solution. The sorptivity index was calculated using Equation (4),

(4) $$S=\frac{F\times d}{M_{SV}-M_{S0}}$$

where ‘*F*’ is the slope of the best fit line between mass gain and the square root of time, average thickness ‘*d*’, *M_SV_*, and *M_S0_* are the vacuum weight and initial weight of samples.

#### 2.2.6. Pore Structure Distribution

Pore structure distribution of all concrete mixtures was measured after 28 days of curing by Autopore 9500 Mercury Intrusion Porosimetry (MIP), (Micrometrics, Ottawa, ON, Canada). After the curing, the samples were collected from each mixture and the hydration was stopped by the solvent exchange method [34]. In this method, samples were exposed for seven days to isopropanol. During this period, the isopropanol was replaced after one day and three days, followed by at least one additional week of vacuum curing. 

**Figure 5:**
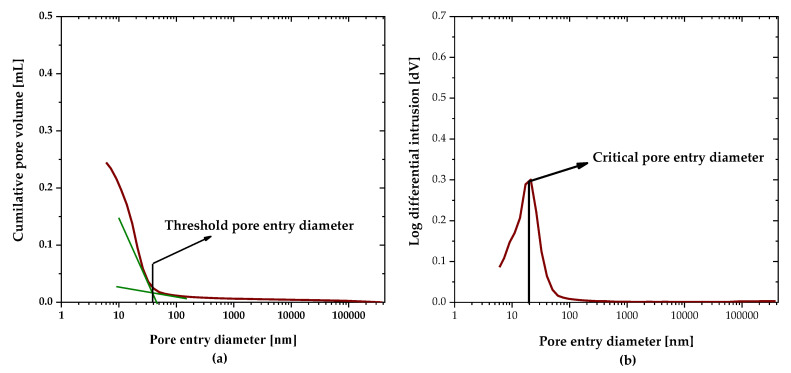
Determination of (**a**) threshold pore entry diameter and (**b**) critical pore entry diameter from MIP data.

The porosimeter is equipped with two different devices, i.e., low pressure and high pressure. The final pressure applied was 206 MPa, which covered the pore entry radius up to 5 nm. The translation of MIP data into pore volume versus pore size was done by use of the Washburn equation [35]. The surface tension of mercury was taken as 0.485 N/m and the contact angle was 130 °C. Two replicates from each mixture were tested. Each mixture’s accessible porosity, threshold, and critical pore entry radius were measured, thereby revealing the pore refinement of each mix. The total mercury penetration converted to each mixture’s accessible porosity. The threshold size of the pore entry diameter is the minimum continuous pore size for the sample as determined by the cumulative volume intrusion curve, and the critical pore size to the peak in the differential curve pore volume indicates the size corresponding to the maximum volume intrusion. Figure 5 illustrates the determination of threshold and critical pore entry diameters from the MIP curves.

## 3. Results

### 3.1. Reactivity

**Figure 6:**
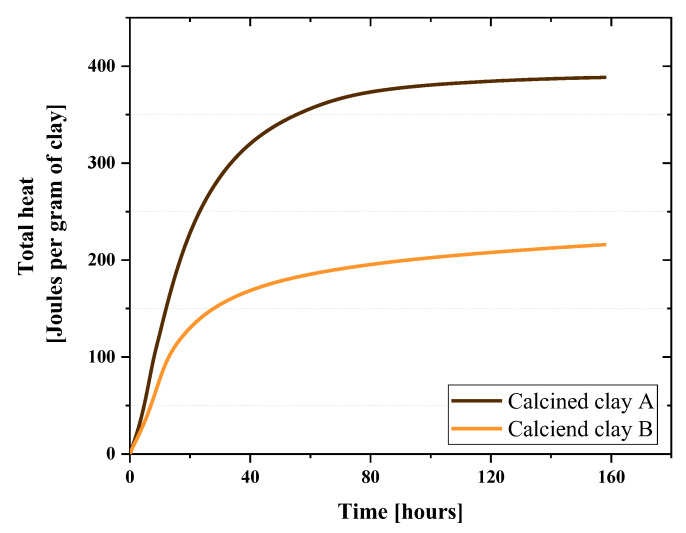
Total heat produced by each clay from R3 test.

Figure 6 illustrates the normalized total heat per gram of clay produced by clay A and B from the reactivity test, respectively. Clay A liberated 388.43 Joule per gram of clay, while clay B was only 215.97 Joule per gram. Therefore, the pozzolanic reactivity of the calcined clay A was 1.79 times more than B in terms of the total heat.

Furthermore, the bound water of these two clays were determined as 10.9% for clay A and 9.4% for clay B. 

### 3.2. Fresh Properteis and Compressive Strength

All the concrete mixtures were targeted for the slump between 90–120 mm. The values of fresh properties are given in Table 5.

**Figure 7:**
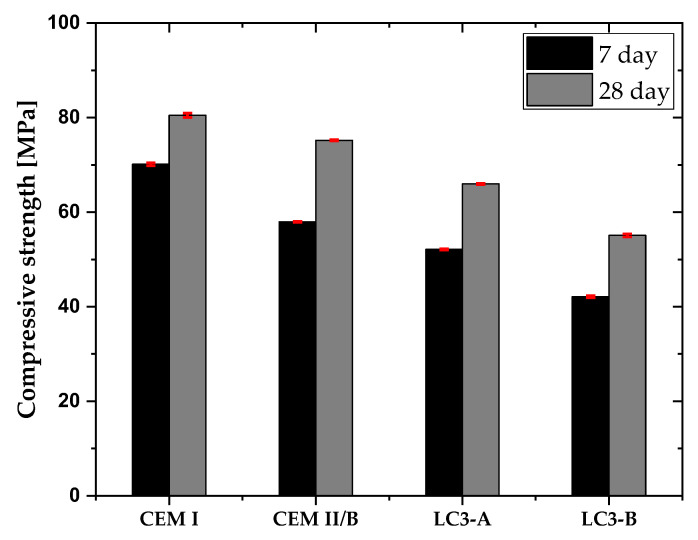
Compressive strength of each mixture.

Figure 7 depicts the compressive strength of each mixture. As anticipated, CEM I produced greater strength than the other combinations. The LC3-A mixture achieved 82% of compressive strength compared to the CEM I mixture after 28 days of curing and 75% of compressive strength compared to the CEM I mixture after seven days. The strength of LC3-B was found to be 69% of the compressive strength of CEM I mix after 28 days of curing.

### 3.3. Water Sorptivity Index

**Figure 8:**
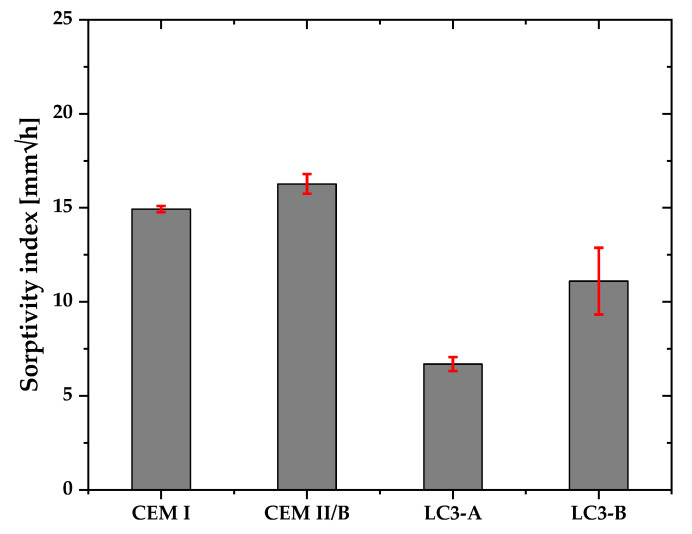
Water sorptivity of each mixture.

Figure 8 depicts the sorptivity index of the concrete samples. The results indicate that CEM I and CEM II/B have a greater sorptivity index than both LC3 mixes. Compared to the Portland cement system, LC3-A was 2.2 times less susceptible to water absorption and 1.25 less than LC3-B. Among clay mixtures, kaolinite content plays an important role. Higher kaolinite content improved the water absorption in LC3 systems. 

### 3.4. Bulk Conductivity and Surface Resistivity

**Figure 9:**
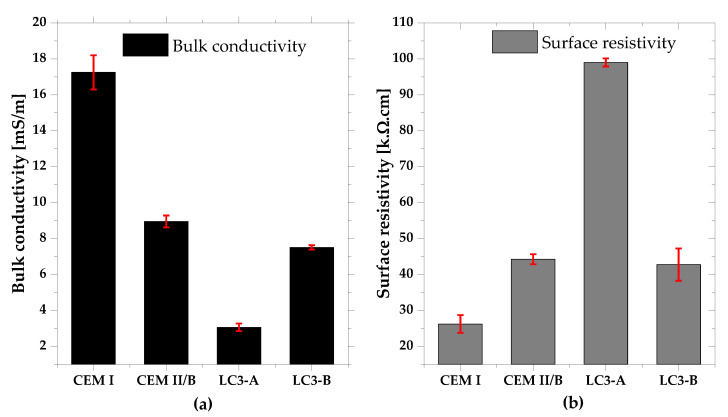
(**a**) Bulk conductivity, and (**b**) Surface resistivity of each mixture.

The electrical conductivity and surface resistivity of each mixture are displayed in Figure 9a,b. Compared to all other mixtures, CEM I exhibited the highest conductivity, whereas LC3-A exhibited the lowest conductivity. As expected, the trend of surface resistivity was just opposite to the conductivity. According to ACI classifications on corrosion rate [36], if the surface resistivity of a combination is greater than 20 k.ohm.cm, it has a negligible risk of corrosion. Thus, both LC3 mixes met this requirement, despite their high replacement levels. Furthermore, the calcined clay’s kaolinite content significantly enhanced the electrical resistance.

### 3.5. Chloride Migration and Chloride Diffusion

**Figure 10:**
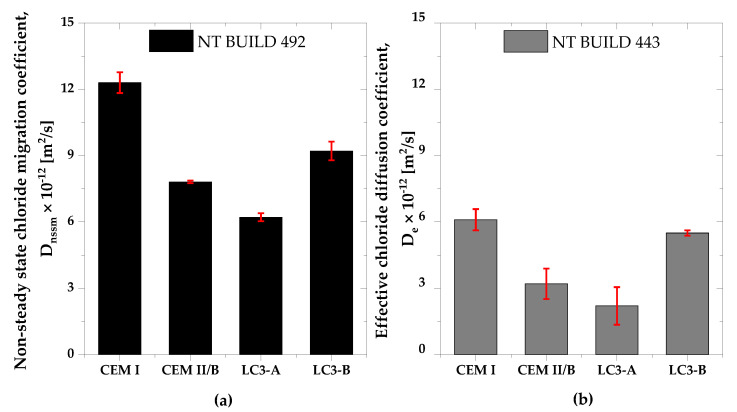
Chloride transport coefficient based on (**a**) NT BUILD 492, and (**b**) NT BUILD 443.

The chloride transport coefficients for each mixture are shown in Figure 10a,b. Both tests showed that the LC3 mixes were resistant to chloride penetration. Even though there was less clinker in LC3-A than in CEM I, the chloride transport coefficients were found to be two-times lower compared to that of CEM I. Additionally, LC3-A outperformed the composite cement CEM II/B. Furthermore, even the LC3-B mix, regardless of a low kaolinite content, performed slightly better than the CEM I mix. 

**Figure 11:**
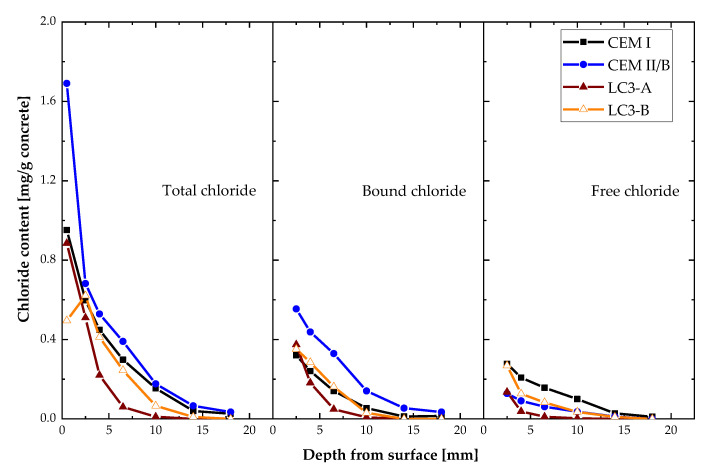
Total chloride content of each mixture categorized into bound and free chloride content.

After evaluating the chloride profile of each mixture, the total chloride content was separated into bound and free chloride contents based on Equation (4). Figure 11 depicts the chloride distribution of each mixture subjected to salt solution. The chloride concentration at each depth reveals that chloride penetration into LC3-A concrete is significantly lower. In addition, the amount of free chloride in the LC3-A mixture was exceedingly low, and most of the chloride was bound. Even the LC3-B mix, which displayed comparable chloride penetration to the mix with CEM I, had a significantly lower content of free chlorides.

**Figure 12:**
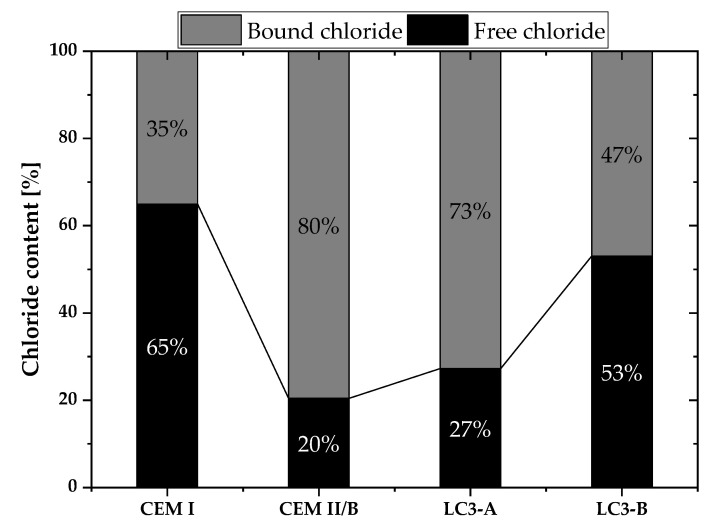
Bound and free chloride content at the depth of 10 mm from exposed surface towards salt solution.

Figure 12 illustrates the quantity of bound and free chloride of all mixtures at a depth of 10 mm from the exposed surface.

As shown in Figure 12, LC3-A contained 73% of bound chlorides, but LC3-B contained only 47%. The calcined clay’s kaolinite content considerably increased the chloride binding capacity of the system. When compared to all other mixes, CEM II/B demonstrated the highest binding capacity, even though the chloride transport coefficient was found to be slightly higher than LC3-A mix.

### 3.6. Pore Size Distribution

**Figure 13:**
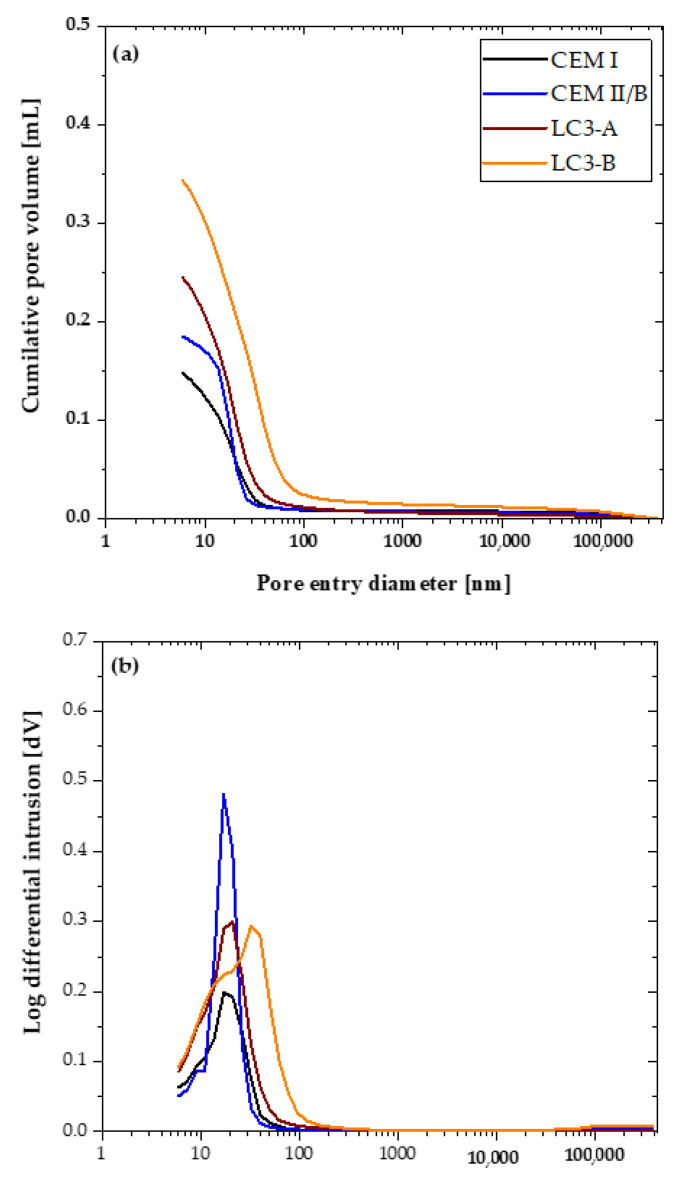
Pore structure distribution of each sample (**a**) total intrusion curve, and (**b**) differential intrusion curve.

**Figure 14:**
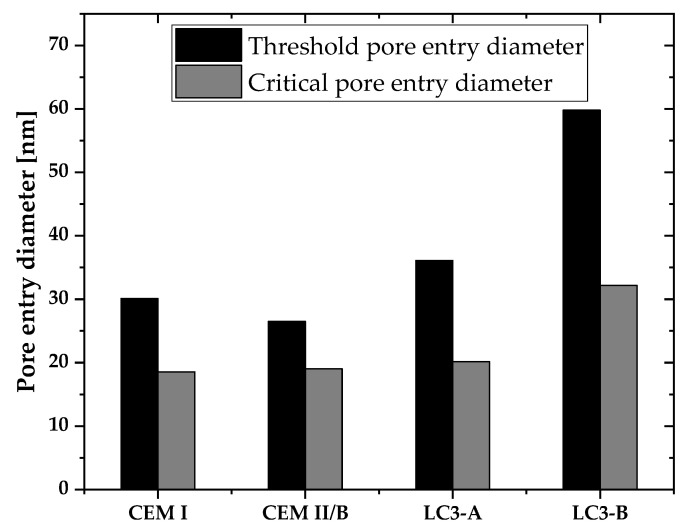
Threshold and critical pore entry diameter of all mixtures.

After 28 days of curing, the pore structure development of each mixture was depicted in Figure 13a,b. The overall porosity of each mixture was determined based on the total mercury intrusion volume. As shown in Figure 13a, LC3-B exhibited a greater degree of porosity than CEM II/B. Moreover, LC3-A exhibited more porosity than CEM I and CEM II/B. In the case of the differential curve, all mixtures except LC3-B exhibited a greater mercury intrusion between 20 and 40 nm. The threshold and critical diameter, derived from cumulative and derivative MIP curves, are depicted in Figure 14. LC3-B demonstrated the greatest critical pore entrance diameter compared to all other mixtures.

**Figure 15:**
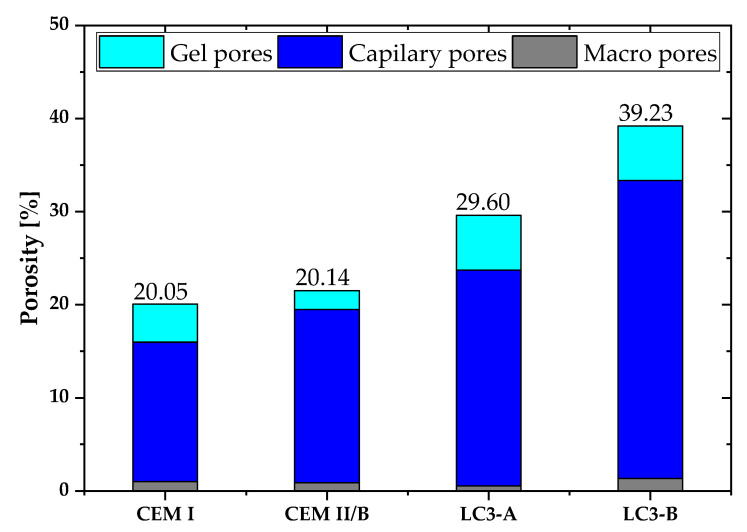
Pore classification based on their entry diameter of each mixture.

Furthermore, the total pore volume of each mixture divided into the classification of macro, capillary, and gel pores based on their entry diameter was analyzed [37]. The different pore categories are illustrated in Figure 15. The quantity of capillary porosity and gel porosity was shown to be significantly greater in the LC3 system, with 83% of LC3-B pores classified as capillary pores. 

## 4. Discussion

The higher reactivity of calcined clay can be directly attributed to the presence of kaolinite [17]; clay with the higher amount of kaolinite shows higher reactivity. This was confirmed by reactivity tests, clearly demonstrating the dependence of reactivity on kaolinite content. In addition, the bound water content of two clays implied that clay A has more reactivity than clay B.

The mix with calcined clay A and calcined clay B achieved 84% and 65% of compressive strength compared to concrete mix with CEM I, respectively. In a blended system, the degree of hydration of the clinker plays a crucial role on the pozzolanic reaction. For example, if the clinker replacement with metakaolin is excessively high, there will not be enough portlandite for the pozzolanic reaction from metakaolin to occur [38,39]. Therefore, even with a higher kaolinite content, there is minimal improvement in compressive strength between clay B and clay A (less than 10 MPa). In comparison to other results reported for the LC3 system [9,10,12], the compressive strength of clay A is somewhat lower. As stated in the section on materials, the clinker itself has a higher alkali content, which might affect the hydration of the LC3 system. The optimal amount of alkali is 0.48 Na2Oeq, but the clinker’s higher alkali content exceeded this limit, which has a slight impact on compressive strength [40]. 

LC3 mix with clay A had greater resistance to capillary absorption than LC3 mix with clay B. In general, the addition of SCMs, along with the combined pozzolanic and filler effects, results in a more complex pore network and a decrease in the water absorption from external environment [9,41]. Figure 7 demonstrates that the LC3 system enhances the resistance against capillary absorption. In addition, the higher kaolinite content enhances the pozzolanic reaction, which might be the cause of less sorptivity index of clay A than clay B. In addition, the bimodal particle size distribution of clay A provides more finer particles than clay B, hence reducing the water absorption through capillary suction [42]. However, LC3-B demonstrated greater resistance to capillary absorption than CEM I and CEM II/B, demonstrating the potential of low-grade clay as an alternative binder in concrete in environments where sorptivity of water is the prevailing durability indicator. 

Compared to other pozzolanic materials used in concrete, the LC3 system is renowned for its higher electrical resistivity and chloride ingress, especially with high kaolinite content. In the case of LC3-A, the surface resistivity was significantly higher than other mixes, which is in accordance with previous research. LC3-B mixes showed comparable resistivity to CEM I mix. The higher surface resistivity of the LC3 system can be attributed to the differences in the kinetics of microstructural development in the binder systems, as well as to the presence of denser microstructure in the LC3 systems [12,43]. The lower reactivity of clay B resulted in the reduction of surface resistivity of the LC3-B mixture. However, the resistivity of LC3-B was still comparable to CEM I. 

In both chloride penetration resistance tests, concrete LC3-A demonstrated superior performance compared to concrete mixes CEM I, CEM II and LC3-B. There are several reasons for this improved behavior, all confirmed in this study and in the literature, which include pore structure refinement, bulk conductivity, chloride binding, and pore solution chemistry [44,45,46,47,48,49,50,51]. The chloride transport coefficients are directly correlated to the surface resistivity, as observed. In addition, the higher kaolinite content contributed to a significant improvement in the development of resistivity, and thus their chloride penetration resistance. Higher discontinuity in the pore network of the LC3 system is attributable to the hydration product and the LC3 system’s reactivity, both of which are evidently related to the kaolinite content [12,43].

After the resistivity, the binding capacity of the LC3 system is considered to be one of the important parameters to influence the chloride penetration [48]. As shown in Figure 12, approximately 74% of the total chloride in LC3-A was bound, whereas LC3-B possessed only 47% of bound chlorides. Maraghechi et al. reported that the amount of Friedel’s salt in the LC3 system is greater than in the Portland cement system containing more than 40 percent kaolinite [43]. This is due to the greater number of carbo-aluminates phases in the LC3 system. The quantity of kaolinite in the clay can therefore contribute to a higher number of carbo-aluminate phases, which can improve chloride binding [9]. However, while comparing the diffusion coefficients of CEM II/B and LC3-A, it was discovered that the impact of chloride binding on chloride transport was minimal. In the case of CEM II/B, there is a greater amount of bound chloride, but the resistance to chloride penetration is not as high as it is for LC-A. The extent of the impact of chloride binding on chloride transport is still debatable, especially in the LC3 systems.

The pore structure of a cementitious system is a well-known parameter which has influence on most of the hardened properties of concrete. In the case of the LC3 system, most of the researchers have reported the better pore refinement compared to other systems. Further, Dhandapani et al. reported that the threshold pore entry diameter and critical pore entry diameter were more refined in the early ages compared to the Portland and fly ash system [10]. Additionally, the conductivity of the systems was linked to the improved pore refinement. However, the percentage of kaolinite in this investigation was nearly 60%; hence, the pore refinement effect of low-grade clay remains unclear. Figure 13a,b demonstrates that the total accessible porosity of MIP was higher than that of the Portland system and CEM II/B. However, the skewness of the differential intrusion curve is lower than that of other systems, indicating the better pore refinement. The number of capillary pores is much higher in LC3-B because of the low reactivity of LC3-B (as shown in Figure 15). If the system has a higher reactivity, the capillary pores will be filled with hydration products at later ages, but this is not possible in the case of a system with a low kaolin content [12], which causes the large number of capillary pores in LC3-B. 

According to several researchers, the quantity of capillary pores affects the chloride penetration in the concrete. But in this investigation, LC3-A, which displayed the lowest chloride transport coefficients, obtained a much higher number of capillary pores. Unlike the capillary pores, the gel porosity obtained in both clays was similar, but the number of macro pores is much higher in LC3-B. Therefore, the influence of gel/capillary porosity on chloride adsorption has yet to be revealed with more scientific evidence. In total, chloride penetration showed higher dependency on the bulk resistivity over chloride binding and pore structure. Finally, the low-grade clay outperformed the ordinary Portland cement system and proved its high potential for use in the concrete. 

## 5. Conclusions

The main aim of the current study was to analyze the durability of LC3 concrete prepared with low- to medium-kaolinitic calcined clay found in East South-East Europe. In the LC3 system, the impact of the alkali content on the development of compressive strength was discovered. The high alkali content weakens compressive strength, especially in early age. Following conclusions are drawn from the study:

- The compressive strength was found to be satisfactory even with low kaolin clay, even though higher kaolinitic clay was needed to attain comparable strength to that of CEM I and blended cement. Concrete with the higher kaolinitic clay was able to achieve around 85% of compressive strength compared to CEM I after 28 days.
- Chloride penetration, electrical activity, and sorptivity of the concrete improved significantly with higher kaolinite content. Chloride diffusion and migration resistance improved by at least 50% compared to Portland cement system with higher kaolinitic clay. Among clays, the chloride penetration resistance differs by 30% and the sorptivity indices value differs by 41%. The kaolinite content was found to be very important in durability performances of LC3 concrete.
- Electric resistivity of the LC3-based concrete dominantly influenced the chloride penetration over the chloride binding and pore structure.
- In the pore structure parameters, the pore refinement (in terms of the critical pore entry diameter) controlled the chloride penetration, while the capillary pore volume was not identified as an important factor in the chloride penetration in the LC3 system in this study.
- Low-kaolinite clay (18% in this study) could be a potential material to make concrete for mild exposure conditions, while higher grade kaolin clay (42% in this study) would be needed for aggressive exposure conditions.

## Figures and Tables

**Table 1:** Chemical oxides of each material used in this study.

Chemical Oxides	CEM I 42.5R	CEM II/B	Clays		Limestone Powder
			A	B	
CaO	63.19	46.94	2.17	2.39	71.59
SiO_2_	19.51	33.65	62.41	61.77	20.21
Al_2_O_3_	4.21	7.55	21.35	28.72	4.32
Fe_2_O_3_	2.85	2.94	7.26	3.03	1.43
MgO	0.85	3.39	1.78	0.68	1.69
Na_2_O	0.20	0.78	1.05	<0.01	0
K_2_O	0.48	0.74	2.50	2.3	0.15
TiO_2_	0.12	0.22	0.94	0.87	0.52
P_2_O_5_	0.45	<0.01	0.36	<0.01	0.42
SO_3_	2.3	4.02	0.07	0.22	1.48
Kaolinite content (%)	--		41.6	18	--

**Table 2:** Mineralogical composition of clinker and clays.

Component	Clinker	Component	Clay-A	Clay-B
C_3_S	56.4 (1)	Quartz	23.2 (4)	21.7 (1)
C_2_S	5.4 (1)	Muscovite	14.6 (1)	27.3 (1)
C_3_A	5.6 (1)	Rutile	0.9 (2)	-
C_4_AF	6.2 (1)	Kaolinite	41.6 (1)	18

**Table 3:** Physical properties of aggregates used in this study.

Property	Fine Aggregate (0/4)	Coarse Aggregate (4/8)	Coarse Aggregate (8/16)
Maximum particle size (mm)	4	8	16
Water absorption (%)	1.3	0.6	0.3
Specific gravity at SSD *	2.79	2.81	2.82

* Surface saturated dry condition.

**Table 4:** Mixture design of all mixtures used in this study.

Mixture	Binder kg/m^3^	w/b	Water, kg/m^3^	Cement, kg/m^3^	Calcined Clay, kg/m^3^	LS kg/m^3^	SP *	Aggregate, kg/m^3^		
							%	8–16	4–8	0–4
CEM I	340	0.40	136	340	-	-	0.7	510	512	1014
CEM II			132	340	-	-	1.2	518	520	1029
LC3-A			136	187	102	51	1.8	513	514	1018
LC3-B			136	340	102	51	1.1	536	538	1064

* wt. % of binder content.

**Table 5:** Fresh properties of each mixture.

Mixture	Paste Volume, L/m^3^	Slump, mm	Temperature, °C	Wet Density, kg/m^3^	Air Content, %
CEM I	246.03	90	24.3	2512.1	3.5
CEM II/B	242.4	105	24.1	2539.9	3.1
LC3-A	261.01	100	23.5	2491.12	3.9
LC3-B	243.65	95	22.3	2395.3	2.8

## Data Availability

Data are available in the manuscript. More detailed information about the data presented in this study are available on request from the corresponding author.
